# Rationale, Design, and Baseline Characteristics of the BioProsthetic Valves with Atrial Fibrillation (BPV-AF) Study

**DOI:** 10.1007/s10557-020-07038-1

**Published:** 2020-07-24

**Authors:** Yutaka Furukawa, Makoto Miyake, Tomoyuki Fujita, Tadaaki Koyama, Misa Takegami, Tetsuya Kimura, Kumiko Sugio, Atsushi Takita, Kunihiro Nishimura, Chisato Izumi, for the BPV-AF Registry group

**Affiliations:** 1 Department of Cardiovascular Medicine, Kobe City Medical Center General Hospital, Kobe, Japan; 2 Department of Cardiology, Tenri Hospital, Tenri, Japan; 3 Cardiovascular Surgery Department, National Cerebral and Cardiovascular Center, Suita, Japan; 4 Department of Cardiovascular Surgery, Kobe City Medical Center General Hospital, Kobe, Japan; 5 Department of Preventive Medicine and Epidemiologic Informatics, National Cerebral and Cardiovascular Center, Suita, Japan; 6 Medical Science Department, Daiichi Sankyo Co., Ltd, Tokyo, Japan; 7 Biostatistics and Data Management Department, Daiichi Sankyo Co., Ltd, Tokyo, Japan; 8 Department of Cardiovascular Medicine, National Cerebral and Cardiovascular Center, Suita, Japan

**Keywords:** Atrial fibrillation, Valvular heart disease, Bioprosthetic valve replacement, Antithrombotic therapy, Thromboembolism, Bleeding

## Abstract

**Purpose:**

To date, clinical data on real-world treatment practices in Japanese patients with atrial fibrillation (AF) after bioprosthetic valve (BPV) replacement are needed. We conducted a large-scale, prospective, multicenter study to understand the actual usage of antithrombotic therapy and the incidence of thromboembolic and bleeding events in these patients, and to eliminate the clinical data gap between Japan and Western countries.

**Methods:**

This was an observational study, in patients who had undergone BPV replacement and had a confirmed diagnosis of AF, with no mandated interventions. We report the baseline demographic and clinical data for the 899 evaluable patients at the end of the enrollment period.

**Results:**

Overall, 45.7% of patients were male; the mean age was 80.3 years; AF was paroxysmal, persistent, or permanent in 36.9%, 34.6%, and 28.5% of patients, respectively. Mean risk scores for stroke and bleeding were 2.5 (CHADS_2_), 4.1 (CHA_2_DS_2_-VASc), and 2.5 (HAS-BLED). Many patients (76.2%) had comorbid hypertension and 54.8% had heart failure. Most BPVs (65.5%) were positioned in the aortic valve. Warfarin-based therapy, direct oral anticoagulant (DOAC)-based therapy, and antiplatelet therapy (without warfarin and DOAC) were administered to 55.0%, 29.3%, and 9.7% of patients, respectively.

**Conclusion:**

Patients enrolled into this study are typical of the wider Japanese AF/BPV population in terms of age and clinical history. Future data accruing from the observational period will contribute to future treatment recommendations and guide therapeutic decisions in patients with BPV and AF.

**Trial registration:**

ClinicalTrials.gov Identifier: UMIN000034485

**Electronic supplementary material:**

The online version of this article (10.1007/s10557-020-07038-1) contains supplementary material, which is available to authorized users.

## Introduction

Globally, it is estimated that 26 million people are affected by heart failure, and the disease places an enormous burden on health care facilities, resulting in more than one million hospitalizations per annum [1]. Valvular heart diseases, including degenerative aortic or mitral valves, or tricuspid valve dysfunction, are some of the most frequent causes of heart failure [2].

The number of operations for valvular heart disease in Japan continues to rise, with almost 12,500 aortic valve operations and 11,000 mitral valve operations performed in 2016 [3]. Among them, bioprosthetic valve (BPV) replacement was estimated to comprise just over 50% of valve replacement operations in 2016, and this is increasing year by year [3]. The concomitant presence of atrial fibrillation (AF) in patients undergoing valve replacement surgery adds additional complexity to treatment decisions [4].

Guidelines published in Europe and the United States consider AF patients with BPV as having non-valvular AF and recommend direct oral anticoagulant (DOAC) treatment [5, 6]. In contrast, Japanese guidelines did not previously classify AF patients with BPV as non-valvular AF [7], but the classification of AF patients with BPV was changed from valvular to non-valvular AF in the recently revised Japanese guideline [8, 9]. However, there is currently insufficient evidence to support these recommendations.

Among the published analyses of several large phase 3 trials comparing DOACs and warfarin in patients with valvular heart disease [10–13], only the ENGAGE AF-TIMI 48 [10] and ARISTOTLE [11] trials enrolled patients with BPV replacements. Unfortunately, the number of BPV cases available for evaluation was small, and although no differences in the incidence of stroke or systemic embolic events and major bleeding with either DOACs or warfarin were reported in either study, more robust clinical evidence is required. In addition, it has been suggested that individuals of Japanese or Asian ethnicity may have more bleeding events and lower rates of thromboembolism compared with Caucasian individuals [14]; thus, the data from the predominantly Caucasian patients in ENGAGE AF-TIMI 48 and ARISTOTLE may not be indicative of the clinical situation in Japan.

Recently, we reported the status and outcomes of patients with BPV replacement and AF in real-world clinical practice [15]. However, that analysis utilized a retrospective, observational design and had a relatively small sample size, and very few patients treated with DOACs were included.

The objective of this study was to collect more up-to-date data on treatment practices by conducting a large-scale, prospective, multicenter study. This will enable us to understand the actual usage of antithrombotic therapy and the incidence of events in patients with AF after BPV replacement in real-world Japanese clinical practice and to eliminate the clinical data gap between Japan and Western countries.

## Methods and Analysis

### Study Design and Assessments

**Fig. 1:**
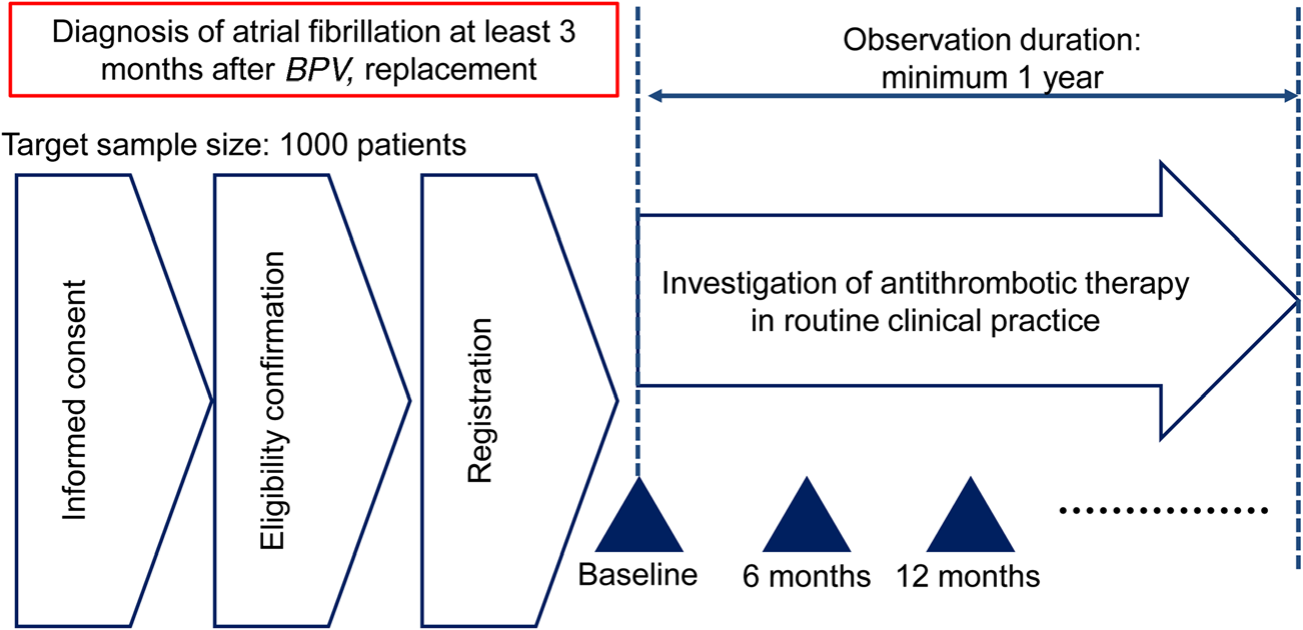
Study design schematic, indicating the stages of enrollment and observation. *BPV*, bioprosthetic valve

This is an ongoing, prospective, multicenter, observational, registry analysis. The study was initiated to understand the actual situation of antithrombotic therapy in daily clinical practice and the occurrence of prespecified efficacy and safety events during the study period. The planned study period is from September 2018 to May 2021. This includes an enrollment period of 1 year (September 2018 to October 2019) and an observation period of a minimum of 1 year (follow-up until October 2020) (Fig. 1). The study was registered at the University hospital Medical Information Network with the identification code UMIN 000034485.

As this is an observational study, no interventions were mandated as part of the study. Evaluation items are shown in Table 1. Observation, examination, and assessment items included patient background, medication administration status (antithrombotic drugs and other agents), invasive surgery status, blood coagulation test data, clinical course and laboratory tests, echocardiography, and adverse events. The risk of stroke was evaluated using the CHADS_2_ and CHA_2_DS_2_–VASc scores, and the risk of bleeding using the HAS-BLED score. These scores were calculated by the investigators using the medical record for each patient. Other background items of interest included smoking and drinking habits, and baseline medical comorbidities and complications. The position and type of BPV, type of surgery performed, and presence or absence of BPV revision were recorded, as was the AF classification (paroxysmal, persistent or permanent), bleeding or thromboembolic history, and all relevant treatment history. For patients receiving warfarin, prothrombin time and international normalized ratio (PT-INR) were measured. Adverse events were recorded using the Japanese Medical Dictionary for Regulatory Activities MedDRA/J version 23.0, along with the severity, outcome, and likely causation.

**Table 1:** Details and timing of items evaluated during the study

Item	Time point during study		
	Registration	Baseline^a^	Every 6 months (± 3 months)
Confirmation of inclusion and exclusion criteria	○		
Consent, visit status, and health check		○	○
Patient characteristics^b^		○	
Antithrombotic drug status		○	○
Administration status of medications other than antithrombotic drugs^c^		○	○
Status of implementation of invasive procedures			○
Blood-clotting test (PT-INR)^d,e^		○	○
Clinical course and laboratory test^e^		○	○
Echocardiography^f^		○	○^f^
Adverse events^g^		Throughout	

*PT-INR*, prothrombin time-international normalized ratio

^a^Baseline was defined as the date of eligibility confirmation and enrollment in the study. If no data were available on the day of baseline, the most recent data were used, with the exception of echocardiogram, which used data immediately after the baseline (i.e., stabilized post-surgery)

^b^Included information on bioprosthetic valve replacement, atrial fibrillation, medical history, and complications/comorbidities

^c^Included antiarrhythmics, antihypertensives, lipid-lowering drugs, antidiabetic agents, treatments for peptic ulcer (proton pump inhibitor, histamine H2 receptor antagonist), and P-glycoprotein inhibitors

^d^Only patients receiving warfarin. The PT-INR data collected included up to the three most recent measurements at baseline and one measurement every 6 months

^e^Data were collected only if implemented under routine clinical practice

^f^Echocardiography was performed every 1 year ± 6 months

^g^Included incidence of stroke, systemic embolism, hemorrhagic event, heart failure requiring hospitalization, and revision of bioprosthesis

The current analysis provides baseline demographic and clinical data for patients included in the study at the end of the enrollment period.

### Patients and Eligibility Criteria

The target population for this study was patients who had undergone BPV replacement and had a confirmed diagnosis of AF. AF was defined as paroxysmal (return to sinus rhythm within 7 days of onset), persistent (persists for more than 7 days after onset), or permanent (electrically or pharmacologically non-defibrillable).

The detailed inclusion criteria were patients who underwent BPV replacement at least 3 months prior to enrollment (either aortic valve or mitral valve position, or both; replacement surgery could be either surgery or transcatheter aortic valve implantation); had a confirmed diagnosis of AF; had at least 1 year of follow-up data during the observation period; and had provided written consent. By requiring at least 3 months between surgery and enrollment, and including the criterion for a confirmed AF diagnosis, patients with transient postoperative AF were excluded. Additional exclusion criteria were participation in or planned participation in any interventional study (to include pharmacologic and non-pharmacologic interventional therapy); moderate or severe mitral stenosis; mechanical valve replacement; and any other reason which meant that participation was judged inappropriate by the investigator. All patients, in either an inpatient or an outpatient setting, who consented to participate in the study, and who met all the inclusion criteria and none of the exclusion criteria, were enrolled in the study.

### Study Endpoints

The study endpoints were defined as the occurrence of each prespecified event during the observation period (event rate). The primary efficacy endpoint was the rate of stroke or systemic embolism, and the primary safety endpoint was the occurrence of major bleeding (based on the International Society on Thrombosis and Haemostasis criteria [16]).

Secondary endpoints included the rates of the following events: stroke; systemic embolism; ischemic stroke; hemorrhagic stroke; intracranial hemorrhage; cardiovascular events (including myocardial infarction, stroke, systemic embolism, and death from bleeding); and bleeding events (including clinically significant bleeding and minor bleeding).

Exploratory endpoints included the rates of the following events: heart failure requiring hospitalization; death from cardiovascular disease (i.e., death undeniably due to cardiovascular causes); all-cause mortality; and revision of bioprosthesis.

### Statistical Analysis

Approximately 900 patients were assumed to be eligible for the study within 1 year of recruitment. In the ENGAGE AF-TIMI 48 trial, the incidence of stroke or systemic embolism event (primary efficacy endpoint) was 1.79% per year [10]. Assuming that the incidence of stroke or systemic embolism (primary efficacy endpoint) was 3% per year in 1000 patients, the 95% confidence intervals (CI) for the data obtained after a mean observation period of 1.0, 1.2, and 1.5 years will be ± 1.1%, ± 1.0%, and ± 0.9%, respectively.

Continuous variables were presented as medians and interquartile ranges (IQR) or means and standard deviations (SD). Categorical variables were recorded as numbers and percentages. In future publications, incidence rates (per 100 patient-years) and 95% CIs of the primary efficacy and safety endpoints during the observation period will be calculated. The cumulative incidence rate of the primary efficacy and safety endpoints will be calculated using the Kaplan–Meier method. The point estimates of the hazard ratio and their 95% CIs will be calculated using the Cox proportional hazard model. The level of significance was set as *p* < 0.05. All statistical analyses were performed using SAS version 9.4 (SAS Institute, Cary, NC, USA).

## Results

Nine hundred and twenty-eight patients were enrolled, of whom 28 patients did not meet the criteria and one patient withdrew. In total, 899 patients formed the analysis set for this study. Of these, 45.7% were male; the mean age was 80.3 years and the mean BMI was 22.2 kg/m^2^ (Table 2). The AF type was paroxysmal in 36.9% of patients, persistent in 34.6%, and permanent in 28.5%. The mean CHADS_2_ and CHA_2_DS_2_-VASc scores were 2.5 and 4.1, respectively, and the mean HAS-BLED score was 2.5. Frequently reported comorbidities (in ≥ 15% of patients) were hypertension (76.2%), heart failure (54.8%), dyslipidemia (49.4%), digestive disease (33.3%), hyperuricemia (25.8%), and diabetes mellitus (21.1%).

**Table 2:** Patient characteristics at baseline, indicating demographic and clinical characteristics

Characteristic	All (*N* = 899)	Warfarin-based therapy (*n* = 494)	DOAC-based therapy (*n* = 263)	Antiplatelet therapy (*n* = 87)	No antithrombotic drugs (*n* = 55)
Male	411 (45.7)	231 (46.8)	105 (39.9)	47 (54.0)	28 (50.9)
Age (years), mean ± SD	80.3 ± 7.0	79.5 ± 6.7	82.4 ± 6.5	79.7 ± 8.1	79.4 ± 8.2
Weight (kg), mean ± SD	53.8 ± 11.4	54.0 ± 11.3	53.4 ± 11.3	54.3 ± 12.1	53.7 ± 11.5
BMI (kg/m^2^), mean ± SD	22.2 ± 3.7	22.1 ± 3.4	22.4 ± 4.2	22.4 ± 3.3	21.8 ± 3.8
CHADS_2_ score					
Mean ± SD	2.5 ± 1.2	2.4 ± 1.2	2.7 ± 1.2	2.4 ± 1.2	2.2 ± 1.1
≥ 2.0	678 (81.0)	362 (78.5)	219 (87.6)	63 (80.8)	34 (70.8)
CHA_2_DS_2_-VASc score					
Mean ± SD	4.1 ± 1.5	4.0 ± 1.4	4.5 ± 1.5	3.8 ± 1.5	3.6 ± 1.5
≥ 3.0	738 (87.8)	397 (85.9)	241 (95.3)	61 (78.2)	39 (81.3)
HAS-BLED score					
Mean ± SD	2.5 ± 1.1	2.5 ± 1.1	2.4 ± 1.0	2.9 ± 1.2	2.1 ± 1.1
≥ 3.0	376 (45.1)	213 (46.5)	101 (40.1)	47 (60.3)	15 (31.3)
eGFR (mL/min/1.73 m^2^)	46.6 ± 17.7	45.5 ± 18.5	48.1 ± 14.8	47.8 ± 20.6	48.3 ± 18.1
Ccr (mL/min)					
Mean ± SD	40.4 ± 18.3	40.2 ± 18.7	40.9 ± 16.4	39.1 ± 21.1	42.3 ± 20.5
Type of AF					
Paroxysmal	332 (36.9)	121 (24.5)	125 (47.5)	56 (64.4)	30 (54.6)
Persistent	311 (34.6)	196 (39.7)	76 (28.9)	24 (27.6)	15 (27.3)
Permanent	256 (28.5)	177 (35.8)	62 (23.6)	7 (8.1)	10 (18.2)
Previous history of CVD					
Ischemic stroke	125 (13.9)	58 (11.7)	49 (18.6)	15 (17.2)	3 (5.5)
Hemorrhagic stroke	21 (2.3)	11 (2.2)	6 (2.3)	3 (3.5)	1 (1.8)
Intracranial hemorrhage	30 (3.3)	13 (2.6)	11 (4.2)	4 (4.6)	2 (3.6)
Systemic embolism	11 (1.2)	7 (1.4)	3 (1.1)	1 (1.2)	0 (0.0)
Major bleeding	47 (5.2)	28 (5.7)	9 (3.4)	6 (6.9)	4 (7.3)
Comorbidities					
Hypertension	685 (76.2)	356 (72.1)	215 (81.8)	74 (85.1)	40 (72.7)
Heart failure	493 (54.8)	275 (55.7)	145 (55.1)	46 (52.9)	27 (49.1)
Dyslipidemia	444 (49.4)	240 (48.6)	135 (51.3)	47 (54.0)	22 (40.0)
Diabetes mellitus	190 (21.1)	110 (22.3)	56 (21.3)	18 (20.7)	6 (10.9)
Renal dysfunction	86 (9.6)	52 (10.5)	15 (5.7)	13 (14.9)	6 (10.9)
Chronic respiratory disease	86 (9.6)	45 (9.1)	31 (11.8)	6 (6.9)	4 (7.3)
Malignant tumor	68 (7.6)	31 (6.3)	27 (10.3)	6 (6.9)	4 (7.3)
Myocardial infarction	45 (5.0)	23 (4.7)	7 (2.7)	13 (14.9)	2 (3.6)
Peripheral arterial disease	33 (3.7)	15 (3.0)	11 (4.2)	2 (2.3)	5 (9.1)
Thrombosis and embolism	28 (3.1)	13 (2.6)	13 (4.9)	2 (2.3)	0 (0.0)
Left ventricular ejection fraction					
< 40%	56 (6.7)	43 (9.4)	6 (2.4)	5 (6.2)	2 (3.9)
40% to 49%	73 (8.7)	50 (10.9)	14 (5.7)	5 (6.2)	4 (7.8)
≥ 50%	709 (84.6)	367 (79.8)	226 (91.9)	71 (87.7)	45 (88.2)

Data are presented as *n* (%) unless otherwise specified

*AF*, atrial fibrillation; *BMI*, body mass index; *Ccr*, creatinine clearance; *CVD*, cardiovascular disease; *DOAC*, direct oral anticoagulant; *eGFR*, estimated glomerular filtration rate; *IQR*, interquartile range; *SD*, standard deviation

### Treatment

The prosthesis was positioned at the aortic valve in 65.5% of patients, at the mitral valve in 22.0% and at both valves in 25.0% (Table 3). Warfarin-based therapy was administered to 494 patients (55.0%), and 263 patients (29.3%) were treated with DOAC-based therapy. Antiplatelet therapy (without warfarin/DOAC) was administered to 87 patients (9.7%) (Table 4).

**Table 3:** Operative characteristics describing the prosthesis position, and full details of the aortic and mitral valves

Characteristic	All (*N* = 899)	Warfarin-based therapy (*n* = 494)	DOAC-based therapy (*n* = 263)	Antiplatelet therapy (*n* = 87)	No antithrombotic drugs (*n* = 55)
Prosthesis position					
Aortic valve	589 (65.5)	259 (52.4)	221 (84.0)	70 (80.5)	39 (70.9)
Mitral valve	198 (22.0)	147 (29.8)	31 (11.8)	9 (10.3)	11 (20.0)
Both valves	112 (12.5)	88 (17.8)	11 (4.2)	8 (9.2)	5 (9.1)
Aortic valve	*n* = 589	*n* = 259	*n* = 211	*n* = 70	*n* = 39
VHD subtype					
Stenosis	445 (75.6)	183 (70.7)	186 (84.2)	50 (71.4)	26 (66.7)
Regurgitation	114 (19.4)	62 (23.9)	28 (12.7)	15 (21.4)	9 (23.1)
Others	30 (5.1)	14 (5.4)	7 (3.3)	5 (7.1)	4 (10.3)
Operation type					
Surgery	352 (59.8)	193 (74.5)	79 (35.8)	49 (70.0)	31 (79.5)
TAVI	237 (40.2)	66 (25.5)	142 (64.3)	21 (30.0)	8 (20.5)
History of replacement					
First replacement	562 (95.4)	242 (93.4)	215 (97.3)	68 (97.1)	37 (94.9)
Re-replacement	25 (4.2)	16 (6.2)	5 (2.3)	2 (2.9)	2 (5.1)
Unknown	2 (0.3)	1 (0.4)	1 (0.5)	0 (0.0)	0 (0.0)
Mitral valve	*n* = 198	*n* = 147	*n* = 31	*n* = 9	*n* = 11
VHD subtype					
Stenosis	84 (42.2)	66 (44.9)	12 (38.7)	2 (22.2)	4 (36.4)
Regurgitation	95 (48.0)	67 (45.6)	18 (58.1)	4 (44.4)	6 (54.6)
Others	19 (9.7)	14 (9.6)	1 (3.2)	3 (33.3)	1 (9.1)
Operation type					
Surgery	198 (100.0)	147 (100.0)	31 (100.0)	9 (100.0)	11 (100.0)
History of replacement					
First replacement	174 (87.9)	127 (86.4)	29 (93.6)	8 (88.9)	10 (90.9)
Re-replacement	24 (12.1)	20 (13.6)	2 (6.5)	1 (11.1)	1 (9.1)

Data are presented as *n* (%)

*DOAC*, direct oral anticoagulant; *TAVI*, transcatheter aortic valve implantation; *VHD*, valvular heart disease

**Table 4:** Administration status of antithrombotic agents (anticoagulant and antiplatelet drugs)

Treatment agent	All (*N* = 899)
No antithrombotic drug	55 (6.1)
Warfarin-based therapy	494 (55.0)
No antiplatelet drug	355
With antiplatelet drug	139
With aspirin (monotherapy)	121
With P2Y_12_ (monotherapy)	12
With DAPT	0
With others	6
DOAC-based therapy	263 (29.3)
No antiplatelet drug	189
With antiplatelet drug	74
With aspirin (monotherapy)	54
With P2Y_12_ (monotherapy)	17
With DAPT	0
With others	3
Antiplatelet therapy (without warfarin/DOAC)	87 (9.7)
Aspirin (monotherapy)	68
P2Y_12_ (monotherapy)	11
DAPT	4
With others	4
Warfarin	*n =* 494
PT-INR	
Age < 70 years	*n* = 27
< 2.0	13 (52.0)
2.0–3.0	12 (48.0)
> 3.0	0 (0.0)
Age ≥ 70 years	*n =* 467
< 1.6	91 (21.6)
1.6–2.6	293 (69.4)
> 2.6	38 (9.0)

Data are presented as *n* (%) unless otherwise specified

*DAPT*, dual antiplatelet therapy; *DOAC*, direct oral anticoagulant; *IQR*, interquartile range; *PT-INR*, prothrombin time-international normalized ratio; *SAPT*, single antiplatelet therapy

## Discussion

This prospective, observational study in 899 patients with AF who have undergone BPV replacement will provide clinically important data to physicians who are currently lacking robust evidence to guide treatment decisions in this patient population. This analysis of baseline data indicates that the patients enrolled into the study are typical of the wider Japanese AF/BPV population in terms of age and clinical history. As such, it is anticipated that future data accruing from the observational period, including the incidence of prespecified thromboembolic and bleeding events, will be widely generalizable and will contribute to future treatment recommendations and guide therapeutic decisions in patients with BPV and AF. In Japan, the validity of considering AF patients with BPV as having non-valvular AF has not been fully explored. Furthermore, clinical study data in this population are limited and come primarily from Caucasian patients [10, 11], which may not reflect the experience in Japanese clinical practice. Data in Japanese patients with AF who have undergone BPV implantation were recently reported from a small-scale retrospective analysis [15], but prospective analyses in the Japanese real-world clinical population are still required to fill the evidence gap. Moreover, although bleeding data have been reported for Asian patients living in the USA [14], it is widely accepted that residential location can have a strong influence on health outcomes (in terms of medical care, environment, and socioeconomic factors). Thus, it is important to observe Asian populations living in Asia to obtain geographically meaningful data.

In comparison with the recent publication from the retrospective BPV-AF registry, in which just 7.5% (16/214) of evaluable patients received DOACs [15], 29.3% (263/899) of patients in our study were found to be treated with DOAC-based therapy at baseline. This difference is likely owing to the timing of the two studies; in the retrospective analysis, we can infer a slow uptake of DOACs in the first few years after approvals were obtained, whereas a higher rate of DOAC prescription is found in current clinical practice. Low numbers of DOAC-treated patients in other AF studies [17–19] have made it difficult to draw accurate conclusions regarding outcomes. Furthermore, another study in which DOACs were administered to a large number of AF patients was restricted to a single area in Japan [20], which can confound the generalization of the data to the wider Japanese population, whereas the current analysis involves multiple study centers across Japan, potentially providing more relevant data for physicians.

Some previous studies have reported that thrombosis and bleeding tendencies differ between Japanese and Western populations [14, 21]. In published phase 3 trials in AF patients, the proportions of Asian patients were low (10.9% to 14.3% in ENGAGE-TIMI 48 [10] and 14.4% to 16.6% in ARISTOTLE [11]). In contrast, in our study, all enrolled patients were Japanese. Thus, our data are expected to suggest appropriate antithrombotic therapy for Asian AF patients.

### Strengths and Limitations

The key strength of this study is the large-scale, prospective, multicenter design, enrolling real-world subjects with BPV, making it reflective of current clinical practice in Japan. We acknowledge that there may be bias inherent in the requirement for 1 year of follow-up data which results in the exclusion of patients with shorter data intervals who may have more severe complications. However, as the study was initiated after DOACs became available in Japan (the first approvals were obtained in 2011 [22]), it will provide the most up-to-date information on the usage of anticoagulants in the real world and deliver a counterpoint to the recent retrospective BPV-AF analysis [15].

Additionally, we did not specifically collect medical history data on percutaneous coronary intervention or coronary artery bypass grafting. Therefore, the risk of coronary events could potentially be underestimated because full information on the presence of coronary artery complications was not available for some patients. However, the CHA_2_DS_2_–VASc score was determined for each patient based on the information in the medical records. While the calculation of the score was dependent on the information in the medical records, we believe that the CHA_2_DS_2_–VASc score is unlikely to be underestimated because it includes complications of coronary artery disease other than a history of myocardial infarction.

## Conclusions

This prospective analysis of 899 patients with AF and BPV replacement in Japanese clinical practice will provide much-needed information to evaluate therapeutic decisions and potential outcomes in this patient population. The baseline data analysis indicates that the study population is typical of AF/BPV patients in Japan, and the outcome data from the observation period are eagerly awaited.

## Electronic supplementary material

ESM 1 (DOCX 16 kb)

## Data Availability

The data that support the findings of this study are available from the National Cerebral and Cardiovascular Center and Daiichi Sankyo Co., Ltd., but restrictions apply to the availability of these data, which were used under license for the current study and so are not publicly available. Data are, however, available from the authors upon reasonable request, and with permission of the National Cerebral and Cardiovascular Center and Daiichi Sankyo Co., Ltd.
