# Supplementary material for: Rationale, Design, and Baseline Characteristics of the BioProsthetic Valves with Atrial Fibrillation (BPV-AF) Study
Source: Cardiovasc Drugs Ther. 2020 Jul 24;34(5):689–96. doi: 10.1007/s10557-020-07038-1 (PMC7497314; doi:10.1007/s10557-020-07038-1)
Supplement: Supplementary file 1 — (DOCX 16 kb) [file 10557_2020_7038_MOESM1_ESM.docx]

# *Cardiovascular Drugs and Therapy*

# Rationale, design, and baseline characteristics of the BioProsthetic Valves with Atrial Fibrillation (BPV-AF) study

Yutaka Furukawa^1^; Makoto Miyake^2^; Tomoyuki Fujita^3^; Tadaaki Koyama^4^; Misa Takegami^5^; Tetsuya Kimura^6^; Kumiko Sugio^6^; Atsushi Takita^7^; Kunihiro Nishimura^5^; Chisato Izumi^8^; for the BPV-AF Registry group

^1^ Kobe City Medical Center General Hospital (Department of Cardiovascular Medicine), Kobe, Japan

^2^ Tenri Hospital (Department of Cardiology), Nara, Japan

^3^ National Cerebral and Cardiovascular Center (Cardiovascular Surgery Department), Osaka, Japan

^4^ Kobe City Medical Center General Hospital (Department of Cardiovascular Surgery), Kobe, Japan

^5^ National Cerebral and Cardiovascular Center (Department of Preventive Medicine and Epidemiologic Informatics), Osaka, Japan

^6^ Daiichi Sankyo Co., Ltd. (Medical Science Department), Tokyo, Japan

^7^ Daiichi Sankyo Co., Ltd. (Biostatistics and Data Management Department), Tokyo, Japan

^8^ National Cerebral and Cardiovascular Center (Department of Cardiovascular Medicine), Osaka, Japan

## Corresponding author

Yutaka Furukawa

Kobe City Medical Center General Hospital (Department of Cardiovascular Medicine), 2-1-1 Minatojima-Minamimachi, Chuo-ku, Kobe 650-0047, Japan

Tel: +81-78-302-4321

Fax: +81-78-302-7537

E-mail: [furukawa@kcho.jp](mailto:furukawa@kcho.jp)

***Online Resource 1 List of investigators and institutions in the BioProsthetic Valves in Atrial Fibrillation (BPV-AF) Registry group***

| **Institution** | **Investigators** |
| --- | --- |
| Division of Cardiology, Department of Internal Medicine, St. Marianna University School of Medicine, Kawasaki, Japan | Masaki Izumo, Ryo Kamijima, Yoshihiro J Akashi |
| Department of Cardiology, Shizuoka City Shizuoka Hospital, Shizuoka, Japan | Yusuke Hattori, Ryuzo Nawada, Tomoya Onodera |
| Department of Cardiovascular Medicine, Kyoto University Graduate School of Medicine, Kyoto, Japan | Takao Kato, Takeshi Kimura |
| Department of Cardiovasucular Surgery, Graduate School of Medicine, Kyoto University, Kyoto, Japan | Kenji Minatoya, Kazuhiro Yamazaki, Hideo Kanemitsu |
| Department of Cardiovascular Medicine, National Cerebral and Cardiovascular Center, Suita, Japan | Chisato Izumi, Masashi Amano |
| Department of Cardiovascular Medicine, Osaka University Graduate School of Medicine, Suita, Japan | Yasushi Sakata, Isamu Mizote, Daisuke Nakamura |
| Department of Cardiovascular Medicine, Kobe City Medical Center General Hospital, Kobe, Japan | Yutaka Furukawa, Takeshi Kitai |
| Department of Cardiovascular Surgery, Kobe City Medical Center General Hospital, Kobe, Japan | Tadaaki Koyama |
| Department of Cardiology, Hyogo Brain and Heart Center, Himeji, Japan | Hiroya Kawai |
| Division of Cardiovascular Medicine, Department of Internal Medicine, Kobe University Graduate School of Medicine, Kobe, Japan | Hidekazu Tanaka |
| Department of Cardiology, Tenri Hospital, Tenri, Japan | Makoto Miyake, Yuki Obayashi, Toshihiro Tamura |
| Department of Cardiology, The Sakakibara Heart Institute of Okayama, Okayama, Japan | Kiyoshi Yoshida, Akihiro Hayashida |
| Department of Cardiology, Kokura Memorial Hospital, Kitakyushu, Japan | Kenji Ando |
| Department of Cardiovascular Surgery, Nagasaki University Hospital, Nagasaki, Japan | Kiyoyuki Eishi |
| Department of Cardiology, Saiseikai Kumamoto Hospital, Kumamoto, Japan | Tomohiro Sakamoto |
| Department of Cardiovascular Medicine, Graduate School of Medical Sciences, Kumamoto University, Kumamoto, Japan | Miwa Ito, Hisanori Kanazawa, Kenichi Tsujita |
| Department of Cardiovascular Surgery, Kumamoto University Hospital, Kumamoto, Japan | Toshihiro Fukui, Hirokazu Tazume |
| Department of Cardiology, Miyazaki Medical Association Hospital, Miyazaki, Japan | Yoshisato Shibata |
| Department of Cardiovascular Surgery, Kurashiki Central Hospital, Kurashiki, Japan | Tatsuhiko Komiya |
